# Severe COVID-19: NLRP3 Inflammasome Dysregulated

**DOI:** 10.3389/fimmu.2020.01580

**Published:** 2020-06-26

**Authors:** Daan F. van den Berg, Anje A. te Velde

**Affiliations:** Amsterdam UMC, Academic Medical Center, Tytgat Institute for Liver and Intestinal Research, Amsterdam Gastroenterology, Endocrinology and Metabolism, Amsterdam, Netherlands

**Keywords:** COVID-19, NLRP3 inflammasome, therapy, endogenous adjuvant activity, HMGB1

## Abstract

SARS-CoV-2 might directly activate NLRP3 inflammasome resulting in an endogenous adjuvant activity necessary to mount a proper adaptive immune response against the virus. Heterogeneous response of COVID-19 patients could be attributed to differences in not being able to properly downregulate NLRP3 inflammasome activation. This relates to the fitness of the immune system of the individual challenged by the virus. Patients with a reduced immune fitness can demonstrate a dysregulated NLRP3 inflammasome activity resulting in severe COVID-19 with tissue damage and a cytokine storm. We sketch the outlines of five possible scenarios for COVID-19 in medical practice and provide potential treatment options targeting dysregulated endogenous adjuvant activity in severe COVID-19 patients.

## Immunopathophysiology of Covid-19

In one of the first analyses of patient characteristics of SARS-CoV-2 infection in Wuhan, China resulting in COVID-19, it was described that the virus affects largely adult age groups. In most patients there is a relative mild course of the infection. However, in 15.7% of affected patients the disease progresses into a severe disease with the need for hospitalization and admission into the ICU (1). Clinically, two phases of immune reaction against the virus can be identified (2). The first phase is the non-severe phase where a specific adaptive immune response is mounted that eliminates the virus and prevents disease progression to a more severe second stage. We will demonstrate from an immunological perspective that it is much more complex and that the body's response to a viral challenge depends on the immune fitness of the person challenged by the viral exposure. The behavior, adaptiveness and responsiveness will determine the intensity, adequacy and magnitude of the response as well as the speed of recovery. These immune fitness parameters can be used to define healthy or deviating behavior of the immune system (3). If the systemic resilience of a person that depends on regulatory subsystems and functional reserves of organs declines, the risks of morbidity and mortality increase (4).

The main question is why most patients show resilience and induce a proper virus eliminating immune response with resolution of the inflammation and what goes wrong in patients that advance to the severe state with tissue damage and an uncontrolled cytokine release, also specified as a cytokine storm.

After the first exposure to a virus, the detection of viral components by the immune system via a number of different receptors on and inside immune cells retinoic acid-inducible gene-I (RIG-I)-like receptors (RLRs), Toll-like receptors (TLRs) and NOD-like receptors (NLRs) and cyclic GMP-AMP synthase (cGAS) activates intracellular signaling cascades, leads to the secretion of type I IFNs and pro-inflammatory cytokines and chemokines (5). Next to generating an innate antiviral response these intracellular signaling cascades also induce expression of co-stimulatory molecules such as CD40, CD80, and CD86 on antigen presenting cells important for initiation of an adaptive immune response. This necessary additional endogenous adjuvant activity is provided by pyroptotic cell death regulated by Nod-like receptor family, pyrin domain-containing 3 (NLRP3) inflammasome activation. These multiprotein complexes form in the cytosol and drive caspase-1 cleavage and the secretion of the pro-inflammatory cytokines IL-1β and IL-18 and other damage-associated molecular patterns (DAMPs) (6). This stimulation of antigen presentation to benefit the induction of an adaptive immune response comes with a cost, because these danger signals give rise to toxicity and are the cause of a rise in body temperature and therefore need to be tightly controlled (7). If not properly monitored, if there is a reduced immune fitness, the consequences can be disastrous with neutrophils infiltrating in tissues, activated macrophages and skewed differentiation of T cells (Th17) all producing pro-inflammatory cytokines resulting in extensive tissue damage.

In the case of the coronavirus SARS-CoV, the endogenous adjuvant activity is caused by the direct activation of NLRP3 by a viral protein, named viroporin protein 3a (8). This viral protein is also present on the genome of SARS-CoV-2 suggesting that SARS-CoV-2 can also directly activate NLRP3 (9). One could ask what the survival/reproduction advantage of inducing NLRP3-mediated pyroptotic cell death would be for the virus, considering the deleterious consequences, including the activation of the immune reaction against the virus and possible death of the host. In contrast to the pyroptotic cell death in human, protein 3a has been described to have a pro-apoptotic function in the original host of the virus: bats (10). Because apoptosis does not, in distinction to pyroptosis, result in an immune reaction, in bats there is dampened immune response when NLRP3 is induced, limiting inflammation and stimulating asymptomatic carriage of the virus (11). So, the direct activation of NLRP3 resulting in pyroptosis could be an unintended side-effect in humans. Given this situation, how can we as humans cope with this activation of NLRP3 by SARS-CoV-2? It is of great clinical relevance to get an answer to this question, because then we might be able to find new markers that predict an outcome and find possible targets for therapeutic intervention that might reduce morbidity and mortality in severe COVID-19. What do we know of the ability to inhibit NLRP3 in patients that seem to be severely affected by SARS-CoV-2?

## Scenarios of Covid-19 Immune Response

**Figure 1:**
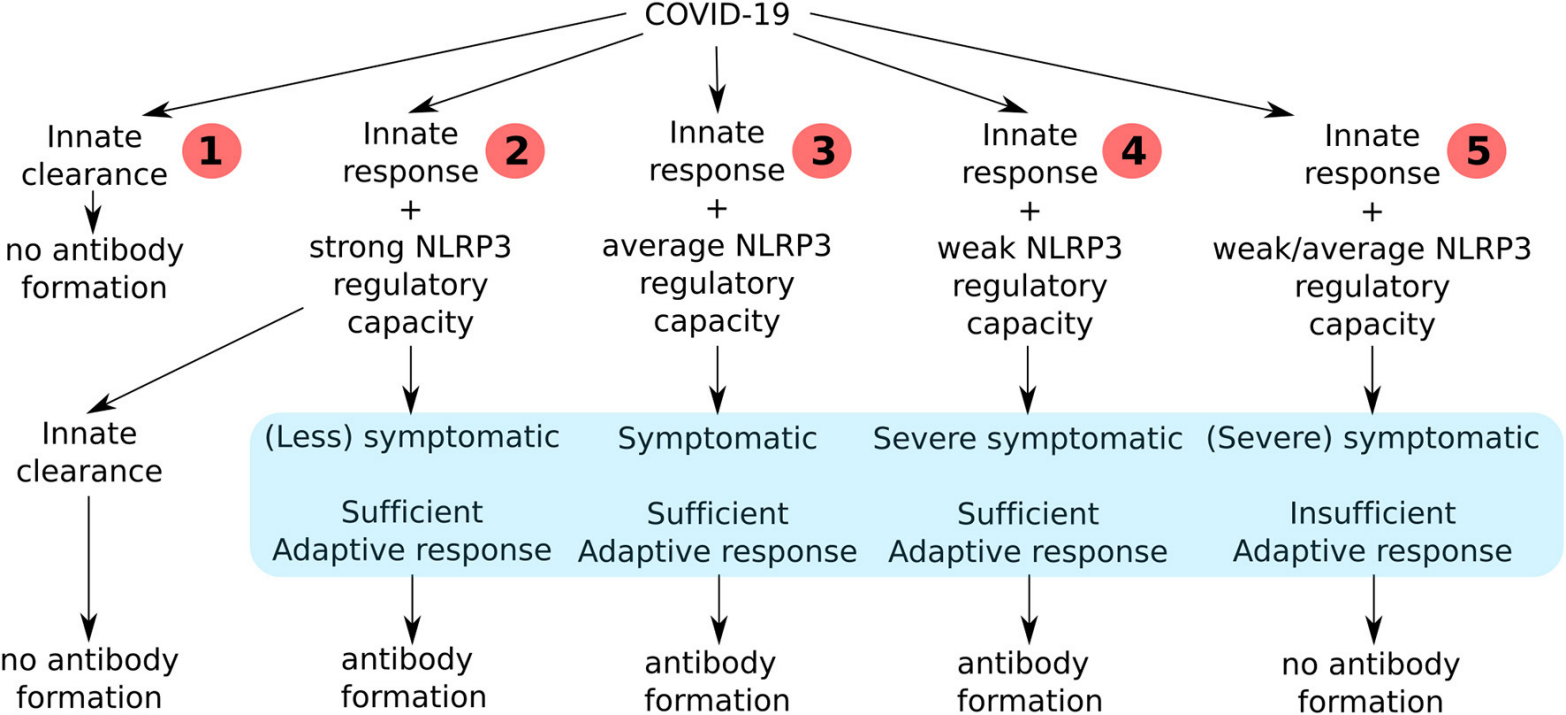
An overview of all the consequences of the clinical course of COVID-19 infection in humans depending on their immune fitness state.

Based on the necessity to tightly regulate NLRP3 and its link to immune fitness there are five possible scenarios to outline the course of the SARS-CoV-2 infection in an individual (Figure 1). In the first scenario, after exposure to low viral load or enough non-specific defense mechanisms the innate immune response will do the job, without the necessity to raise an adaptive immune response. In this scenario there is lysis and phagocytosis by NK cells and macrophages, enough to clear all infected cells. The inflammatory activation of these cells is low and does not pass the threshold needed to activate NLRP3. In some cases it can nonetheless be activated coinciding with weak to average symptoms but not followed up by an adaptive response. In the second scenario there is NLRP3 activation that is strongly downregulated after the initial co-stimulation necessary for APC activation followed by a sufficient adaptive response and production of antibodies against the virus. In the third scenario there is some systemic effect resulting in clinical symptoms like fever and sickness behavior (12) because of the cytokines released during NLRP3 activation that is subsequently downregulated followed by a sufficient adaptive response and antibody production. In the fourth scenario a sustained NLRP3-dependent inflammatory response results in severe clinical symptoms, necrosis, DAMP release and severe inflammation of the lungs. During a period of severe illness the patient is eventually able to mount an adaptive response with antibody production and recovers. In the fifth scenario the innate response is not able to clear the infection, resulting in an NLRP3 activation that is useless because the patient is unable to mount an adaptive response leading to viral clearance (13). In people that have a reduced capacity to mount a protective immune response it is possible that the virus will propagate and massive destruction of affected tissues will occur. This will lead to more DAMPs and a vicious circle of NLRP3 activation will finally result in death.

In all of our scenarios there is a central role for NLRP3 inflammasome regulation. Most literature is focused on the hyper activation of the NLRP3 inflammasome and the detrimental effect of the release of endogenous danger signals on the host. As already stated, the inflammation needs a tight control to be able to restore homeostasis after a challenge of the immune system. The downregulation of the NLRP3 inflammasome can be regulated in different ways, by post-translational modification of the NLRP3 inflammasome or by different NLRP3-interacting regulators. The post-translational modification of NLRP3 inflammasome can be mediated by ubiquitination or phosphorylation (14, 15). NLRP3-interacting regulators Pyrin-only proteins (POPs) and CARD-only proteins (COPs) function in the downregulation of the inflammation. Expression of some of the POPs is upregulated by NF-κB and IL-1β resulting in a feedback loop to prevent excessive NLRP3 activation (16). The COPs bind caspase-1 preventing autoactivation and limiting NLRP3 inflammasome activation (17).

**Figure 2:**
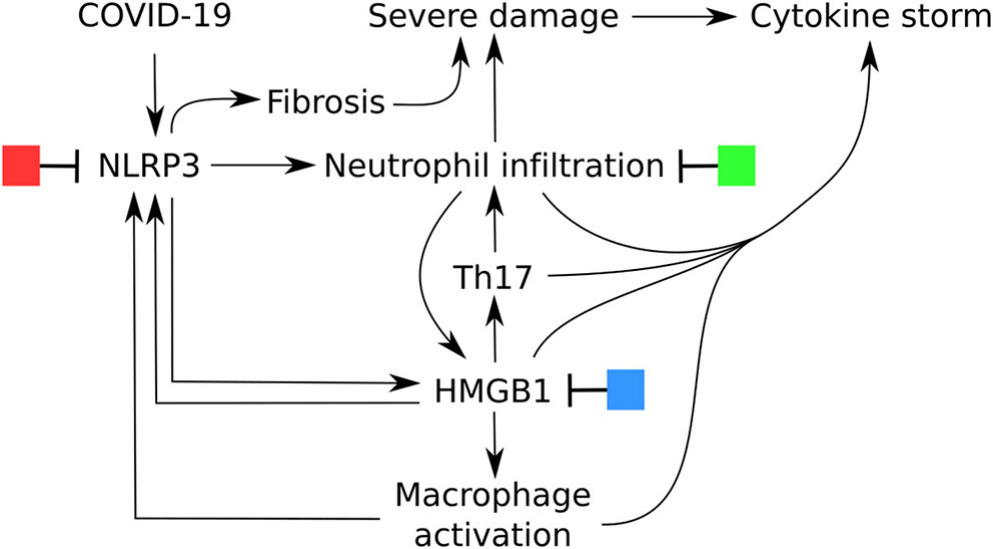
Central role of NLRP3 inflammasome activation in the severe symptomatic phase of COVID-19 and potential options for treatment.

The DAMPs released after NLRP3 inflammasome activation have a dual function. In a normal immune reaction they induce the necessary co-stimulatory activation of the APC, but they also play a role in resolution and tissue regeneration. Only in case of a hyperactivation of the NLRP3 inflammasome DAMPs are released in high concentrations and result in pyroptosis, High mobility group box 1 (HMGB1) release, activation of macrophages, neutrophil infiltration and reduced apoptosis, excessive cytokine production (IL-1β, IL-2, IL-6, IL-17, TNF-α, G-CSF, GM-CSF, IFN-γ, CXCL10, CCL2, and CCL3, cytokine storm) and fibrosis (Figure 2) (18–22). Not only does it explain the diversity of the symptoms of the patients, but it might also explain heterogeneity in the affected patients. Male PBMC were found to express significantly higher mRNA levels of NLRP3 pathway-related genes NLRP3, ASC (PYCARD), CASP1, CASP5, and IL1B (all *P* < 0.0001) than female PBMC (23). Moreover, patients where the most lethality is observed are elderly and patients with non-communicable diseases and obesitas (1). Elderly patients having an “inflammaging,” a low grade inflammation associated with NLRP3 inflammasome priming and activation and weaker inhibition (24) and, obese patients with a metainflammation (25) resulting in a higher base activity of NLRP3 (26, 27). An enhanced exposure to DAMPs and NLRP3 inflammasome activation can affect immune fitness and is the result of a complex interplay where genetics [SNPs in NLRP3 (28)] and also lifestyle factors [such as exercise, reduce NLRP3 activation (29), certain diets, block or stimulate NLRP3 activation (30, 31) and, air pollution, induces NLRP3 activation (32)] are interconnected.

In search for a pathway to relate sustained NLRP3 inflammasome activation in aging we found a microRNA that has Pyrin-only protein 1 (POP1) as its target (33). This miR-34-5p is found to be increased in skeletal muscle and in serum-derived extracellular vesicles in an experimental model and considered as an “inflammiR” (34). From these data it is tempting to speculate that age-increased miR-34-5p results in the diminished capacity to deactivate NLRP3 by inhibiting POP1 production.

Evidence is accumulating that one of the main downstream DAMPs of NLRP3 activation is HMGB1. HMGB1 was originally discovered to be involved in endotoxin lethality in mice (35). It is a critical late marker of sepsis (36) and infection responsible for epithelial barrier failure, organ dysfunction, vascular leakage and even death (37). In high levels HMGB1 is a central mediator of an excessive inflammatory response and severity of pathology during the course of viral infections (7, 38), but low levels mediate sickness behavior, antibacterial activities and might be beneficial when accelerating alveolar epithelial repair (39). Most of the evidence comes from experimental influenza virus models and acute lung injury where infection/injury induces increased HMGB1 levels in the lungs that contribute to the severity of pneumonia, correlate to death and can be blocked with HMGB1-specific antibody (38, 40). This increased HMGB1 is also responsible for neutrophil infiltration, regulated via IL-17 (41). Taken together, overactive NLRP3 with neutrophil infiltration, Th17, HMGB1 and macrophage activation is likely to be the cause for the pathological findings and the cytokine storm in severe COVID-19 (42, 43), which is hyperstimulated by positive feedback loops (44).

## Treatment Options

The discrimination into two phases of the clinical disease requires also the need for a dual treatment approach (2). In the first immune defense-based protective phase there is a need for therapies that reduce virus entry and help to eradicate the virus by boosting the immune system. In the second inflammation-driven damaging phase the endogenous adjuvant reaction of the immune system should be suppressed. In Figure 2 there are potential options for treatment depicted. For each of these options a large number of potential candidates are available. We will highlight some and refer to other authors that have summarized this. The first clinical study for a NLRP3 inflammasome inhibitor (Tranilast) to treat COVID-19 is ongoing and registered in the Chinese clinical trial registry (45). Other studies are still in a pre-clinical phase and study the effect on acute lung injury or on cell lines for example with resveratrol (46), tetracycline (47) or erythropoietin (48) or nicardipine, a L-type calcium antagonist (49), lidocaine (50) CP-456,773 (51), Diacerein (52). For colchicine it is hypothesized that it has an effect on NLRP3-mediated diseases (53). In several reviews other NLRP3 inflammasome inhibitors are listed (14, 16, 27, 54).

A second potential target for treatment is HMGB1 (55). In experimental models of acute lung injury or sepsis blocking of HMGB1 or one of its receptors has shown a beneficial effect (38, 40, 56, 57). Even though the anti-HMGB1 has no effect on the proliferation of the virus, in combination with peravimir a significant effect on neutrophil infiltration and macrophage aggregation was observed (57). Also Chloroquine (58), Methotrexate (59), anti-oxidants (60–62), traditional Chinese medicine (63, 64), thrombomodulin (65), and others (66–68) are listed as potential therapeutic strategies to diminish HMGB1.

Another option to limit severe damage would be to reduce the number of neutrophils. Already in a phase II clinical trial for COVID-19 CM4620-IE is tested^1^ This is a calcium release-activated calcium CRAC channel inhibitor aiming to stabilize pulmonary endothelial capillary barrier, reduce neutrophil infiltration and prevent lung injury (69). Several candidates from pre-clinical work can be distinguished, Galactin-9 inhibits the infiltration of neutrophils and decreases MMP levels and moreover down-regulates Th1 and Th17 T cells (70) and exogenous carbon monoxide delivered from carbon monoxide-releasing molecule 2 inhibits neutrophil infiltration (71). This treatment also inhibited NLRP3 activation *in vitro* (72) and HMGB1 in an *in vivo* model (73) and a suggestion is made that this could also be of use in the current ICU (74).

Finally, also blocking the downstream mediators of NLRP3 inflammasome activation caspase-1 and cytokines IL-1β and IL-18 and their receptors are potential options for treatment for COVID-19-related pneumonia (75–77).

## Final Remarks

The data presented in this overview suggest that the NLRP3 inflammasome with its downstream pathways is an attractive target for therapy of COVID-19 with (severe) pathology in individuals that have a low immune fitness. Knowledge of early indications of possible scenarios after infection will be needed to be able to timely intervene with an appropriate therapy. Several potential candidates are available that are already or might be readily tested in clinical practice. For prevention early signaling of the presence of low grade inflammation might be an indicator for loss of resilience leading to vulnerability to a viral challenge. It also might be an incentive to implement lifestyle changes to enhance immune fitness.

## Author Contributions

DB: performed literature search, figures, data collection, data analysis, and data interpretation. AV: performed literature search, study design, data collection, data analysis, data interpretation, and writing. All authors contributed to the article and approved the submitted version.

## Conflict of Interest

The authors declare that the research was conducted in the absence of any commercial or financial relationships that could be construed as a potential conflict of interest.

## Footnotes

^1^https://www.globenewswire.com/news-release/2020/04/09/2014265/0/en/FDA-Grants-CalciMedica-Permission-to-Begin-Dosing-CM4620-IE-in-Patients-with-Severe-COVID-19-Pneumonia-under-a-Newly-Opened-IND.html
